# Trends in suicide mortality among prostate cancer survivors in the United States, 1975–2019

**DOI:** 10.1186/s12889-023-17589-1

**Published:** 2024-01-05

**Authors:** Hao Wan, Xiangpeng Zhan, Situ Xiong, Tao Chen, Xiaoqiang Liu, Xinxi Deng, Songhui Xu, Bin Fu

**Affiliations:** 1 Department of Urology, The First Affiliated Hospital, Jiangxi Medical College, Nanchang University, Nanchang, Jiangxi Province China; 2 Department of Urology, Jiu Jiang No. 1 People’s Hospital, Jiujiang, Jiangxi Province China

**Keywords:** Prostate cancer, Suicide mortality, SEER, Trend, United States

## Abstract

**Background:**

Suicide was an important cause of death in prostate cancer. This study intended to investigate trends in suicide mortality among prostate cancer (PCa) survivors from 1975 to 2019 in the United States.

**Method:**

We identified PCa survivors from the Surveillance, Epidemiology, and End Results (SEER) program from January 1975 to December 2019. Standardized mortality rate (SMR) was calculated d to assess the relative risk of suicide in PCa survivors compared with the general men population. Poisson regression model was performed to test for trend of SMRs. The cumulative mortality rate of suicide was calculated to assess the clinical burden of suicide mortality.

**Results:**

7108 (0.2%) cases were death from suicide cause, and 2,308,923(65.04%%) cases recorded as dying from non-suicidal causes. Overall, a slightly higher suicide mortality rate among PCa survivors was observed compared with general male population (SMR: 1.15, 95%CI: 1.09–1.2). The suicide mortality rate declined significantly relative to the general population by the calendar year of diagnosis, from an SMR of 1.74(95%CI: 1.17–2.51) in 1975–1979 to 0.99(0.89–1.1) in 2015–2019 (*Ptrend* < 0.001). PCa survivors with aged over 84 years, black and other races, registered in registrations (including Utah, New Mexico, and Hawaii) failed to observe a decrease in suicide mortality (*Ptrend* > 0.05). The cumulative suicide mortality during 1975–1994 was distinctly higher than in 1995–2019(*P* < 0.001).

**Conclusion:**

The trend in suicide mortality declined significantly from 1975 to 2019 among PCa survivors compared with the general male population in the United States. Notably, part of PCa survivors had no improvement in suicide mortality, and additional studies in the future were needed to explore it.

**Supplementary Information:**

The online version contains supplementary material available at 10.1186/s12889-023-17589-1.

## Introduction

Prostate cancer (PCa) was the second leading cause of cancer-related death among men in the United States, with 268,490 newly diagnosed cases in 2022 [1, 2]. Despite the improved survival rate of PCa in the United States, there has been an alarming increase in the cause of death for PCa [3]. It is increasingly believed that longevity does not guarantee a good quality of life [4]. It is becoming increasingly evident that longevity alone does not guarantee a good quality of life. In fact, studies have suggested that PCa survivors may face a higher risk of suicide compared to the general population, attributed to factors such as anxiety associated with cancer diagnosis, pre-existing psychosocial conditions, and poor quality of life resulting from treatment side effects [5–7]. For instance, A study by Katja Fall et al., on Swedish males aged 30 years or older diagnosed with prostate cancer, found an increased risk of cardiovascular events and suicide in newly diagnosed males with prostate cancer [6]. One statistical data presented that the suicide rates of the general population and cancer-specific population in the United States were 16.7 and 31.4 per 100,000 person-years, respectively [8]. A study on veterans showed that they were significantly affected by preexisting posttraumatic stress disorder (PTSD), and it was found that depression, substance use disorder, and any specific prostate cancer treatment served as partial mediators [9]. Some developed European countries have also observed a similar situation [6, 10]. In addition, some statistics on the causes of death of PCa also show that suicide is an important cause of death of PCa patients [11, 12].

In recent years, there has been a shift in the mode and concept of treating PCa as we gain a better understanding of cancer diseases. The recognition that cancer and its treatment can have a negative long-term impact on quality of life was first demonstrated by the National Cancer Act in the United States in 1971 [13]. Since then, rehabilitation programs aimed at improving the quality of life of PCa survivors have emerged, potentially reducing suicidal tendencies among this population. However, it is important to note that the existing research data may be outdated and may not accurately reflect the current suicide mortality rates among PCa survivors. Therefore, the aim of this study is twofold. Firstly, we seek to analyze the changing trends in the suicide mortality rate of PCa survivors compared to the general population in the United States over the past 45 years. Secondly, we aim to determine if, based on the latest data, we still observe a higher suicide mortality rate among PCa survivors compared to the general population.

## Methods

### Data source

We conducted a population-based cohort study using data from the Surveillance, Epidemiology, and End Results (SEER) program spanning from 1975 to 2019. This retrospective, population-based cohort study was based on the SEER-8 prostate cancer survivor database and a reference cohort from U.S. mortality data. The data of SEER-8 prostate cancer survivors came from the case list of *Incidence - SEER Research Plus Data, 8 Registries, Nov 2021 Sub (1975–2019)*, which covered approximately 8.3% of the U.S. population (based on the 2010 census) and contained information of cancer incidence, treatment, and survival. The National Center for Health Statistics (NCHS) of the Centers for Disease Control and Prevention (CDC) took charge of U.S. mortality data, and the cause of death and population data were determined according to the death certificate.

### Study population

We identified patients diagnosed with PCa as the first primary malignant tumor from SEER-8 registrations (Atlanta, Utah, Seattle, New Mexico, Iowa, Hawaii, Connecticut, San Francisco-Oakland SMSA) between January 1, 1975, and December 31, 2019 (Supplement Fig. 1). We excluded prostate cancer patients whose diagnosis age was less than 20 or with unknown survival time, race is, and cause of death. Finally, a total of 3,549,972 prostate cancer patients were identified. Then, we collected the following data of PCa patients: age at diagnosis, race (white, black, others including American/Indian/Alaska/Native, Asian/Pacific Islander), year of diagnosis, marital status [married; single; separated/divorced/widowed (SDW)] household income, tumor stage (localized/regional, distant, unknown) surgery data, radiotherapy data, cause of death and survival month.

**Fig. 1:**
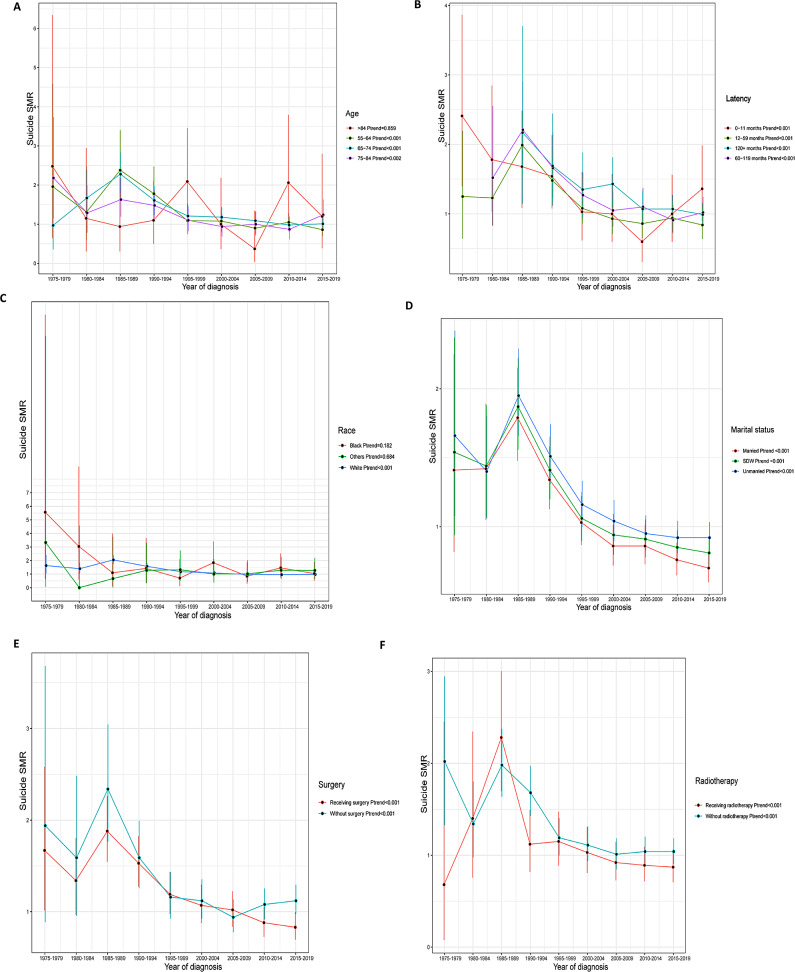
Trend in suicide SMR among prostate cancer survivors by (**A**) age at diagnosis; (**B**) latency; (**C**) race; (**D**) marital status; (**E**) surgery; (**F**) radiotherapy

### Suicide mortality and follow-up

The SEER program recorded cause of death (COD) with the International Classification of Diseases (ICD), version 9 from 1979 to 1998, and version 10 were utilized to record patients who died after 1998. Suicide cause death was coded E950–E959 in 1979–1998 based on ICD-9 and U03, X60–X84, and Y87.0 based on ICD-10. For convenient analysis, we classified the causes of death of PCa patients as suicide and non-suicide cause. The time of follow-up in this study started at the time of diagnosis of PCa. and ended at the date of death, last recorded as alive, and last follow-up (December 31, 2019), whichever came first. The minimum unit of follow-up time was a month.

### Statistical analyses

To assess the relative risk of suicide in prostate cancer survivors compared with the general men population, we used SEER*Stat version 8.3.8 to calculate the standardized mortality rate (SMR; the ratio of observed suicide deaths in prostate cancer survivors to expected deaths in the general male population) and 95% confidence intervals (CIs), adjusted for age at death, race, and year of death. The number of expected deaths was calculated by multiplying the suicide mortality rate of the general men population in the U.S. by the cumulative follow-up time in the study cohort.

We explored trends in suicide SMRs by calendar year of prostate cancer diagnosis (increase by five years), age of diagnosis (increase by five years), and latency after prostate cancer diagnosis (0–11 months, 12–59 months, 60–119 months, 120 + months). Poisson regression model was performed to test for trend of SMRs, and it set expected death cases as the offset. The statistical results were exhibited as coefficient and *Ptrend*, representing the direction and significance of Poisson regression model, respectively. Subgroup analysis was conducted to determine the trend of suicidal SMR with year of diagnosis by stratifying the study population by age, race, treatment (surgery and radiotherapy), marital status, and registration. In order to assess the clinical burden of suicide mortality, we calculated the cumulative mortality rate of suicide (and the corresponding 95% CIs) and took into account the competitive death risk of non-suicide causes [14]. We calculated the cumulative suicide mortality rates of 5 years and 10 years by calendar year of prostate cancer diagnosis, respectively. The competition risk model was performed by R and RStudio Version 4.6.0.0(utilizing *cmprsk* package), and *Ptrend* tests were conducted in Stata version 14 (StataCorp, College Station,TX).

Two-sample t-tests and Chi-square tests were applied for continuous and categorical variables, respectively. All results were 2-sided, and *P* < 0.05 was considered statistically significant.

## Results

### Descriptive characteristics of study population

Of all identified PCa survivors, there were 7108 (0.2%) cases of death from suicide cause, and 2,308,923(65.04%%) cases were recorded as dying from non-suicidal causes (Table 1). Among PCa patients in five age groups: <55, 55–64, 65–74, 75–84, and ≥ 85, the number of suicides were 1855 (26.1%), 1693 (23.8%), 2206 (31.0%), 1146 (16.1%), and 208 (2.9%) respectively. The non-suicidal patients numbered 352,367 (15.2%), 480,103 (20.8%), 694,199 (30.1%), 567,945 (24.6%), and 214,309 (9.3%). In the case of prostate cancer patients belonging to the White, Black, and Other racial groups, the proportions of suicides were 93.2% (6623 cases), 2.2% (158 cases), and 4.6% (327 cases) respectively. The remaining patients, who did not die by suicide, constituted 85.4% (1,970,953 cases), 7.1% (163,889 cases), and 7.5% (174,081 cases) respectively. During the research period of 1975–1984, 1985–1994, 1995–2004, and 2005–2019, the proportion of PCa patients who died by suicide were 1536 (21.6%), 2022 (28.4%), 1786 (25.1%), and 1764 (24.8%) respectively. The number of non-suicidal patients were 504,167 (21.8%), 620,420 (26.9%), 592,082 (25.6%), and 592,254 (25.7%) respectively. For married, single, and SDW PCa patients, the percentages of suicides are 2578 (36.3%), 3985 (56.1%), and 545 (7.7%) respectively. Among patients with household incomes of < $35,000, $35,000-$54,999, $55,000-$74,999, and $75,000+, the number of suicides were 25 (0.4%), 698 (9.8%), 1963 (27.6%), and 1883 (26.5%) respectively. For prostate cancer patients with tumor stage Localized/regional and Distant, the number of suicides were 4113 (57.9%) and 760 (10.7%) respectively. The mean age of suicide cases was relatively lower than that of non-suicide cases (62.3 vs. 67.8; *P* < 0.001). Survivors who died from suicide had a significantly longer survival time than non-suicide cases (mean 77.3 vs. 64.7, *P* < 0.001; median 48.0 vs. 25.0, *P* < 0.001).

**Table 1:** Overall characteristics of prostate cancer survivors by survival outcome, 1975–2019

	Alive (N = 2,308,923)	Suicide cause (N = 7108)	Non-suicide cause (N = 1,233,941)	Total (N = 3,549,972)
**Age (year)**				
**Mean (SD)**	56.8 (14.4)	62.3 (14.4)	67.8 (12.9)	64.0 (14.4)
**Median [Min, Max]**	57.5 [17.5, 85.0]	67.5 [17.5, 85.0]	67.5 [17.5, 85.0]	67.5 [17.5, 85.0]
< 55	502,596(40.6%)	1855 (26.1%)	352,367 (15.2%)	856,818 (24.1%)
55–64	344,125 (27.9%)	1693 (23.8%)	480,103 (20.8%)	825,921 (23.3%)
65–74	272,246 (22.1%)	2206 (31.0%)	694,199 (30.1%)	968,651 (27.3%)
75–84	96,995 (7.9%)	1146 (16.1%)	567,945 (24.6%)	666,086 (18.8%)
≥ 85	17,979 (1.5%)	208 (2.9%)	214,309 (9.3%)	232,496 (6.5%)
**Race**				
White	1,014,269 (82.2%)	6623 (93.2%)	1,970,953 (85.4%)	2,991,845 (84.3%)
Black	93,658 (7.6%)	158 (2.2%)	163,889 (7.1%)	257,705 (7.3%)
Others^a^	126,014 (10.2%)	327 (4.6%)	174,081 (7.5%)	300,422 (8.5%)
**Year of diagnosis**				
1975–1984	30,538 (2.5%)	1536 (21.6%)	504,167 (21.8%)	536,241 (15.1%)
1985–1994	87,146 (7.1%)	2022 (28.4%)	620,420 (26.9%)	709,588 (20.0%)
1995–2004	238,814 (19.4%)	1786 (25.1%)	592,082 (25.6%)	832,682 (23.5%)
2005–2019	877,443 (71.1%)	1764 (24.8%)	592,254 (25.7%)	1,471,461 (41.5%)
**Marital status**				
Married	371,430 (30.1%)	2578 (36.3%)	919,607 (39.8%)	1,293,615 (36.4%)
Single	750,665 (60.8%)	3985 (56.1%)	1,279,031 (55.4%)	2,033,681 (57.3%)
SDW	111,846 (9.1%)	545 (7.7%)	110,285 (4.8%)	222,676 (6.3%)
**Household income**				
< $35,000	4028 (0.3%)	25 (0.4%)	6018 (0.3%)	10,071 (0.3%)
$35,000-$54,999	125,150 (10.1%)	698 (9.8%)	198,708 (8.6%)	324,556 (9.1%)
$55,000-$74,999	489,347 (39.7%)	1963 (27.6%)	611,602 (26.5%)	1,102,912 (31.1%)
$75,000+	551,940 (44.7%)	1883 (26.5%)	691,175 (29.9%)	1,244,998 (35.1%)
Unknown	63,476 (5.1%)	2539 (35.7%)	801,420 (34.7%)	867,435 (24.4%)
**Tumor stage**				
Localized/regional	763,578 (61.9%)	4113 (57.9%)	1,087,200 (47.1%)	1,854,891 (52.3%)
Distant	48,290 (3.9%)	760 (10.7%)	525,128 (22.7%)	574,178 (16.2%)
Unknown	422,073 (34.2%)	2235 (31.4%)	696,595 (30.2%)	1,120,903 (31.6%)
**Surgery record**				
Undergo surgery	312,875 (25.4%)	4356 (61.3%)	1,178,289 (51.0%)	2,103,711 (59.3%)
Without surgery	312,875 (25.4%)	2752 (38.7%)	1,130,634 (49.0%)	1,446,261 (40.7%)
**Radiotherapy record**				
Receiving radiotherapy	395,453 (32.0%)	1997 (28.1%)	659,614 (28.6%)	1,057,064 (29.8%)
Without radiotherapy	838,488 (68.0%)	5111 (71.9%)	1,649,309 (71.4%)	2,492,908 (70.2%)
**Survival month**				
Mean (SD)	130 (110)	77.3 (83.9)	64.7 (85.9)	87.3 (99.9)
Median	103	48.0	25.0	48.0

SD: standard deviation; SDW: separated, divorced or widowed;

a: others included American Indian, Alaska Native, Asian, and Pacific Islander

### The trend in Suicide SMR by calendar year of PCa diagnosis

Overall, we observed a slightly higher suicide mortality rate among PCa survivors when compared with general male population (SMR: 1.15, 95%CI: 1.09–1.2). However, the suicide mortality rate of PCa survivors declined significantly relative to the general population by the calendar year of PCa diagnosis, from an SMR of 1.74(95%CI: 1.17–2.51) in 1975–1979 to 0.99(0.89–1.1) in 2015–2019 (*Ptrend* < 0.001) (Table 2).

**Table 2:** Trend in suicide mortality by calendar year of diagnosis, age at diagnosis and latency after PCa diagnosis among PCa patients

	SMR	95%CI
**Overall**	1.15	1.09–1.2*
**Calendar year of diagnosis**		
1975–1979	1.74	1.17–2.51*
1980–1984	1.41	1.08–1.8*
1985–1989	2.01	1.72–2.34*
1990–1994	1.56	1.35–1.78*
1995–1999	1.18	1.02–1.35*
2000–2004	1.1	0.96–1.25
2005–2009	0.98	0.86–1.11
2010–2014	0.98	0.88–1.1
2015–2019	0.99	0.89–1.1
*Ptrend* (Coefficient)	< 0.001 (-0.091)	
	**SMR**	**95%CI**
**Age at diagnosis (year)**		
45–49	0.72	0.2–1.85
50–54	0.94	0.6–1.41
55–59	0.96	0.74–1.23
60–64	0.98	0.81–1.18
65–69	1.16	1.01–1.33*
70–74	1.24	1.11–1.38*
75–79	1.13	1.01–1.25*
80–84	1.16	1.04–1.29*
85+	1.20	1.08–1.33*
*Ptrend* (Coefficient)	0.078(0.023)	
	**SMR**	**95%CI**
**Latency after PCa diagnosis (month)**		
0–11	1.26	1.09–1.46*
12–59	1.11	1.02–1.2*
60–119	1.17	1.07–1.27*
120+	1.13	1.04–1.24*
*Ptrend* (Coefficient)	0.599(-0.012)	

SMR: standardized mortality rate, CI: confidence interval, PCa: prostate cancer,

*: statistical significance

**Fig. 2:**
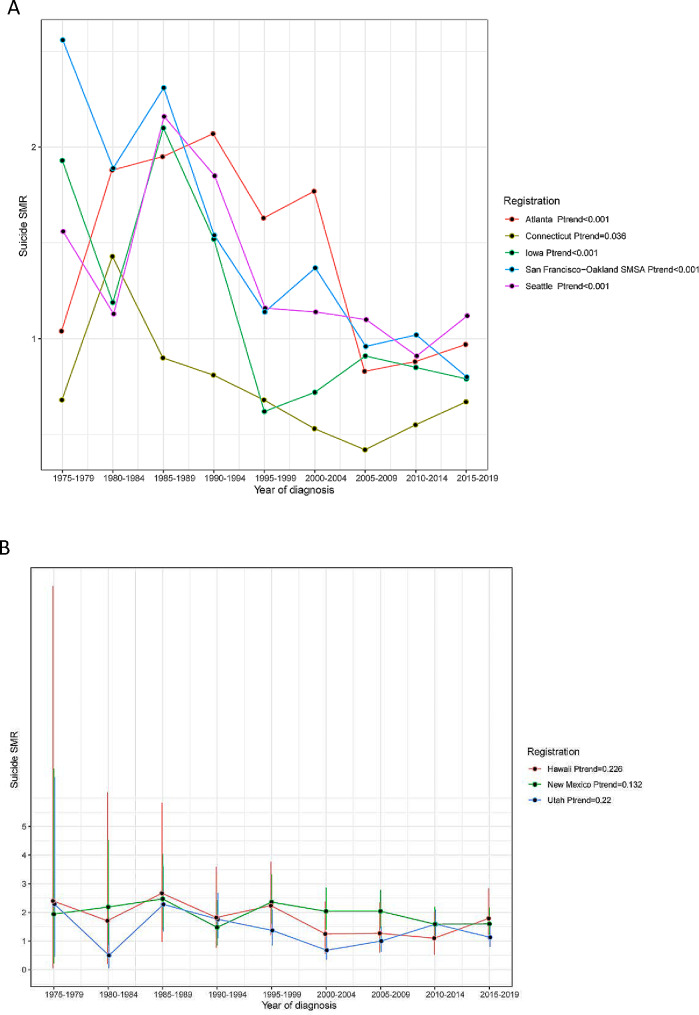
Trend in suicide SMR among prostate cancer survivors by registration

In the subgroup analysis stratified by the age of diagnosis, we observed a statistically significant decline in suicide SMR among PCa survivors aged 55–64(*Ptrend* < 0.001), 65–74(*Ptrend* < 0.001), 75–84(*Ptrend* = 0.002), respectively (Fig. 1A). Of PCa survivors aged over 84, we failed to obtain a statistically significant trend (*Ptrend* = 0.858). We observed a similar decline in suicide SMR at 1 year, 5 years, 5 to 10 years, and more than 10 years after the diagnosis of prostate cancer (All *Ptrend* < 0.05) (Fig. 1B). Suicide SMR among white PCa survivors declined by year of diagnosis (*Ptrend* < 0.001), while the trend in black (*Ptrend* = 0.182) and other races (*Ptrend* = 0.684) was not significative (Fig. 1C). The trend in suicide SMR decreased significantly when we stratified the study population by marital status (all *Ptrend* < 0.001) (Fig. 1D). This study showed a similar decline trend in suicide SMR among PCa survivors who have received or have not received surgery and radiotherapy (all *Ptrend* < 0.001) (Fig. 1EF). Of eight registrations enrolled, there were five registrations (included Atlanta, Seattle, Iowa, Connecticut, San Francisco-Oakland SMSA) that showed a decreasing trend in suicide SMR (all *Ptrend* < 0.05) (Fig. 2A), and the other three registrations (included Utah, New Mexico, Hawaii) did not show this declining trend (all *Ptrend* > 0.05) (Fig. 2B).

### The trend in suicide SMR by age and survival time

Suicide SMR increased slightly with the age of prostate cancer survivors, from an SMR of 0.72(95%CI: 0.2–1.85) in the 45–49 age group to 1.20(95%CI: 1.08–1.33) in the 85 + age group. However, this trend does not show statistical significance (*Ptrend* = 0.078) (Table 2). Simultaneously, we also failed to obtain a statistically significant trend in suicide SMR by latency after PCa diagnosis (*Ptrend* = 0.599) (Table 2).

### Cumulative mortality of suicide

**Fig. 3:**
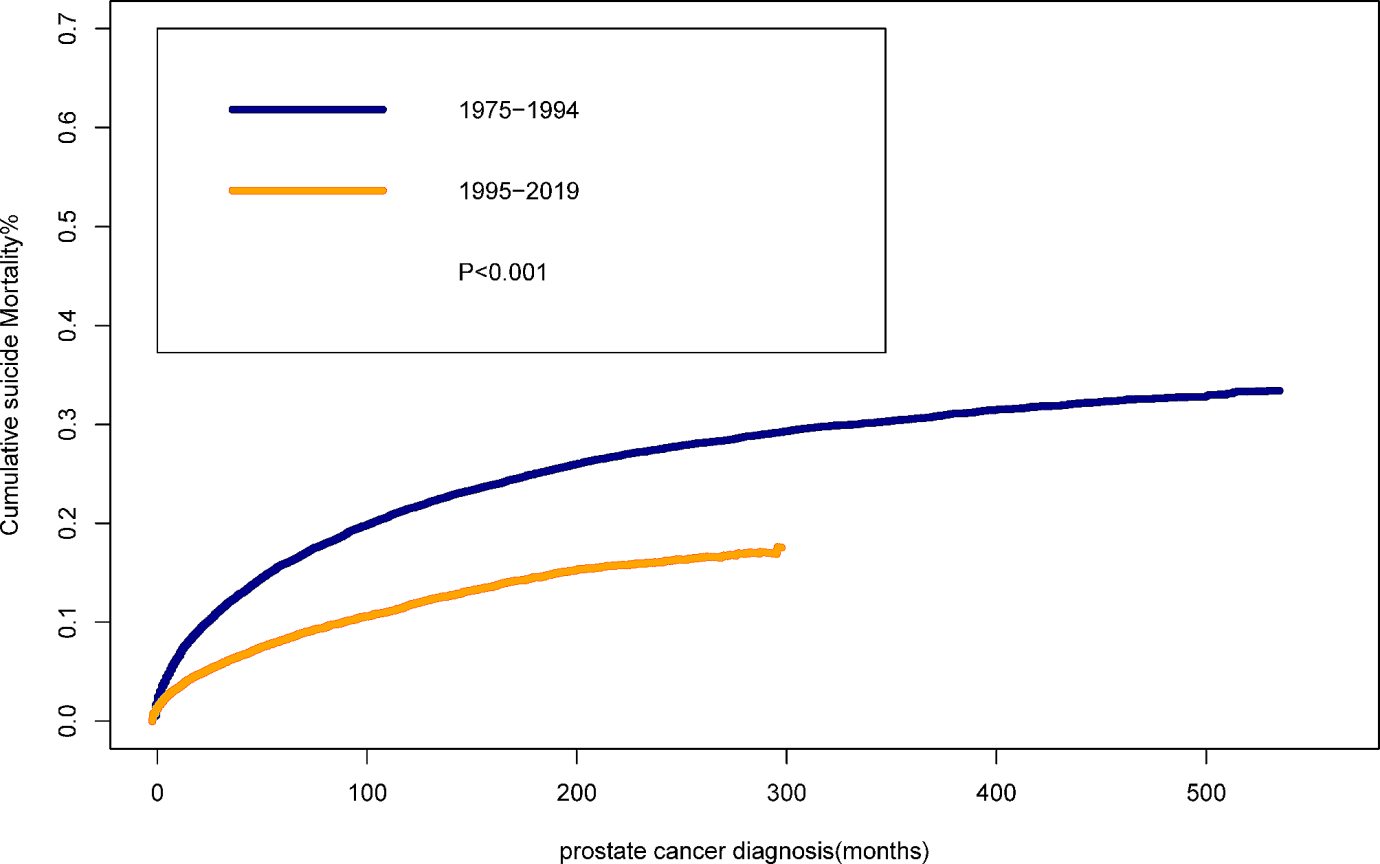
Cumulative mortality for suicide among prostate cancer survivors by year of diagnosis

Cumulative mortality analysis showed that the clinical burden of suicide was different in the calendar year of diagnosis of PCa survivors (Fig. 3). The cumulative suicide mortality of PCa survivors diagnosed in 1975–1994 was distinctly higher than that in 1995–2019(*P* < 0.001). The 5-year cumulative mortality of PCa survivors diagnosed in 1975–1994 decreased from 0.14 to 0.09% in 1995–2019. In addition, the 10-year cumulative mortality declined from 0.2% in 1975–1994 to 0.13% in 1995–2019.

## Discussion

In this study, we found a steady decline in suicide mortality from PCa compared with the general male population in the United States from 1975 to 2019. According to the data from 2015 to 2019, there was no statistical difference in suicide mortality between PCa survivors and the male general population in the United States.

In PCa survivors, the effects of the disease itself and cancer treatment on survivors’ health were well known, including the existence of poor prognosis, the degree of disease progression, the existence of depressive symptoms, complications caused by surgery, complex hormones, sexual dysfunction and so on [13, 15]. These factors can all have detrimental effects on the health of patients. Despite the significant public health concerns surrounding the gun and opioid epidemics during this period, the instances of suicide related to these issues remain relatively low among cancer survivors. In contrast, the overall rates of suicides involving guns and drugs have risen within the United States population, indicating substantial differences in the effects of these epidemics and the risk factors for suicide between cancer survivors and the general population [16]. Overall, suicidal intention in PCa survivors was associated with both physical and psychological disorders [17–19]. For instance, PCa survivors were more likely to experience additional chronic diseases like arthritis, heart disease, diabetes, asthma, and osteoporosis than the general population [20]. From a psychological point of view, a meta-analysis of data showed that clinically related depression accounted for 17%, 15%, and 18%, respectively, before, during, and after PCa treatment [7]. These physical and mental disorders caused by prostate cancer were essential explanations for the high suicide mortality rate of PCa survivors in previous studies [8, 10, 21].In addition, prostate cancer not only affects erectile dysfunction caused by the disease itself, but also includes mental illnesses caused by cancer or its treatment. Prostate cancer itself reduces libido and frequency of sexual intercourse. Furthermore, surgical or hormonal treatments that block testosterone further increase the frequency of erectile dysfunction [22, 23]. Similarly, post-treatment penile cancer has a profound impact on the quality of life, as there is an increase in postoperative patients’ depression and sexual anxiety. This is a significant factor contributing to a high suicide rate [24].

We observed a declining trend in suicide mortality among PCa survivors compared with the general male population in a longer-term follow-up. This declining trend seemed not surprising after considering our further understanding of disease and improving its treatment model. For example, the concept of cancer as a long-term or chronic disease had gradually taken root in people’s hearts and has been incorporated into many cancer treatment guidelines [13, 23]. Prostate cancer was particularly suitable for this concept of long-term treatment because it contributed to a large number of long-term cancer survivors [25, 26]. The National Cancer Act, introduced in the United States in 1971, confirmed this idea and began implementing various measures to improve cancer survivors’ long-term quality of life [27]. They believe that there was a need to establish a new model of comprehensive cancer rehabilitation, including a multidisciplinary team of providers aimed at optimizing the physical, psychological, occupational, and mental health of survivors, taking into account the limitations caused by the chronic or late effects of cancer treatment and other complications. Simultaneously, to recognize the unique psychosocial pressure on cancer survivors, the National Cancer Institute established the Office of Cancer survivors in 1996, whose grand mission is to minimize or stabilize the adverse effects of cancer survivors [16].In addition, The implementation of the Garrett Lee Smith Memorial Act in 2004 [28] and the Joshua Omvig Veterans Suicide Prevention Act of 2007 [29] greatly reduced healthcare costs associated with emergency visits and hospitalizations. A body of evidence suggested that these measures were effective in improving the quality of life of PCa survivors. For instance, a Cochrane systematic review proved that psychosocial intervention for PCa survivors could improve their quality of life and reduce pain and depression [30]. Similarly, changes in diet and physical activity have been shown to improve the quality of life and health outcomes of PCa survivors [31]. What we found seemed to exhibit a good result of these measures was that the negative effect of prostate cancer on survivors had been reduced, although we still lacked such follow-up data. Meanwhile, due to the widespread adoption of prostate-specific antigen (PSA), an increasing number of PCa patients were being detected at an early stage, preventing these patients from suffering more serious disease [32]. This might relatively reduce the psychological and physical burden of PCa patients.

Meanwhile, we still needed to note that in some subgroups, including age over 84, the race of black or others, and registrations (including Utah, New Mexico, and Hawaii), we did not find a significant decline trend in suicide mortality among PCa survivors. It is not easy to make a reasonable explanation for this result. Previous studies have indicated that elderly male cancer survivors have a comparatively higher risk of suicide compared to other segments of the population. This is mainly attributed to the fact that older men tend to swiftly transition from suicidal ideation to actual attempts [27, 33]. This might be a crucial reason why there was no significant decrease in suicide mortality among PCa survivors over 84 in this study. Notably, we found that suicide mortality increased with age among PCa survivors, even if we did not get a meaningful *Ptrend*. Therefore, relatively elderly PCa survivors might be more worthy of attention in improving their long-term quality of life. Strengthening the long-term quality of life of PCa survivors who had no significant improvement in suicide mortality might be a challenge for improving the care system for PCa survivors in the future.

This study provided the first comprehensive analysis of the trend of suicide mortality from PCa over the past 45 years and sent a good signal that with the development of psychological oncology care, palliation, and hospice care, there has been a significant decrease in suicide mortality in PCa survivors [8, 16, 34]. This study had several limitations that need to be noted. First, this was a retrospective nature with unavoidable selection bias. Second, although we had identified a group of PCa survivors who had no improvement in suicide mortality because we lacked follow-up data after the diagnosis of PCa, it was difficult for us to make a reasonable explanation and give suitable clinical guidance. More work in the future was needed to explore it to decrease their suicide mortality. Then, some important information such as whether suicide occurred before or after treatment, rehabilitation programs, hormone therapy, mental illnesses and criminal/legal problems were lacking from the SEER database. In addition, we have to admit that given such a large amount of population information, there might be misclassification of the cause of death in the SEER database. However, this indiscriminate misclassification was unlikely to deviate from the trend. Finally, what was inconsistent with previous studies was that we had not found a significant trend in suicide mortality with latency after PCa diagnosis. For example, Stephnie Misono et al. found suicide mortality of cancer survivors was the highest five years after cancer diagnosis [8]. This might be due to a relatively long study period in this study, so the trend of suicide mortality was not noteworthy.

## Conclusions

The trend in suicide mortality declined significantly from 1975 to 2019 among PCa survivors compared with the general male population in the United States. Recent data showed no significant difference in suicide mortality between PCa survivors and the general male population. Notably, part of PCa survivors had no improvement in suicide mortality, and their psychological experience in the future deserved further attention, especially considering the measures taken now had made a positive effect.

### Electronic supplementary material

Below is the link to the electronic supplementary material.

**Supplement Figure 1**: Flowchart of Prostate Cancer patients incorporation from SEER database.

## Data Availability

The data in this article comes from the SEER database This data can be found here: https://seer.cancer.gov/data/.
