# Supplementary material for: Trends in suicide mortality among prostate cancer survivors in the United States, 1975–2019
Source: BMC Public Health. 2024 Jan 5;24:101. doi: 10.1186/s12889-023-17589-1 (PMC10770994; doi:10.1186/s12889-023-17589-1)
Supplement: Supplementary file 1 — Supplement Figure 1: Flowchart of Prostate Cancer patients incorporation from SEER database. [file 12889_2023_17589_MOESM1_ESM.docx]

Supplement Figure 1: Flowchart of Prostate Cancer patients incorporation from SEER database.
